# Therapeutics of the Throat: Æsculus Hipp.; Ailanthus Gland.

**Published:** 1888-02

**Authors:** John V. Allen


### THERAPEUTICS OF THE THROAT.

### Æsculus Hippocastanum.

#### THROAT, OBJECTIVE.

Congestion of fauces, of palate, of tonsils.
Dryness, of mucous surfaces of.
Inflammation of tonsils.
Mucus, hawks up ropy.
" " " " with sweetish taste.
" " " thick.
" " " " afterward watery.
" frequent calls to expectorate.
" in, excites cough.
Swollen, worse evening, and on swallowing.
Swelling of tonsils, worse, left tonsil, swallowing.

#### THROAT, SUBJECTIVE.

Aching, constant, in tonsils and soft palate.
Biting, pain in fauces and tip of tongue.
Burning, violent, with raw feeling.
" and *dry* sensation of fauces and palate.
" in œsophagus and mouth.
" like fire, worse swallowing.
Burnt, as if.
Colder, sensation as if *air* breathed in were.
Contraction, sensation of.
Constricted, scraped sensation of the, causing disposition to hawk.
Constriction of the fauces, great, with tongue feeling as if it had been scalded.

Dryness of the, worse after eating.
" " " painful.
" " " as if scraped and swollen.
" " " posterior part of.
" " " with scraped sensation, worse during expectoration.
" " " with sweetish taste.
" " fauces and œsophagus.
" " with frequent desire to swallow.
Excoriated and scraped, felt as if.
Formication, violent in the.
Fullness in upper part of, sensation of.
Hot, felt worse left side.
Pricking and dull pressing in, with sensation of fullness in epigastrium, with empty eructation, followed by burning in stomach and bowels.
Pressure in the pit of, as if something had stuck there which required to be expelled.
Pain, sharp, in fauces and tip of tongue.
" increased in, worse after eating a grape.
Raw feeling in.
Roughness and dryness of, as from taking cold.
Swallow, frequent inclination to.
" " " " with dull pains in tonsils.
" " " " " neuralgic pains in tonsils.
" " " " " constant aching in tonsils and fauces.
" " " " " severe constricted feeling in fauces.
" " " " " feeling of dryness and stiffness of throat when swallowing.
" " " " from feeling as if something had lodged in fauces.
Swallowing difficult.
Scraped, sensation as if, exciting cough.
Shooting, constant in the.
Soreness of, worse forenoon, worse evening and swallowing.
Tickling in, causing cough.

AGGRAVATIONS.

Eating, after.
" " grapes, pain.

Expectoration, during, dryness of throat.
Evening in the, soreness.
Forenoon in the, soreness.
Side, left, hot feeling.
Swallowing, burning, soreness.

CONCOMITANTS.

A careful study of its pathogenesis would lead us to believe that it affected the whole mucous membranes in a peculiar manner, marked congestions of a venous type are excited, particularly of the veins in the lower rectum; the peculiar dryness produced by this remedy is most marked in the mucous membrane lining the alimentary canal, especially those of the throat and rectum. We have hæmorrhoidal tumors, which protrude from the rectum, and are of a blue purple color, with sharp, shooting, cutting pains in them running *up* the rectum; with this we have the dry, uncomfortable feeling in the rectum, which feels as if it had been filled with *sticks*. The hæmorrhoids are large, without much hæmorrhage, and with the dryness and itching is associated a feeling of heat.

Pain across the sacro-iliac symphysis, more or less constant, of a dull, aching character, with a feeling as if the back would give way at that point; worse when walking or stooping; this is an important symptom, and may be regarded as a key-note in uterine displacements.

DIFFERENTIATION.

Æsculus is one of the many drugs which presents few peculiarities in its array of throat symptoms, therefore, we have few comparisons to make. The dryness, as you know, is very marked, and is made worse by *eating*. Now *Nat. mur.* should be carefully compared, as both have *dryness*, worse *eating;* and in *Nat. mur.* he constantly hawks transparent mucus, with the dryness of throat; this is not so with Æsculus, if he hawks the mucus is *ropy* in character.

The tonsils, which are smooth, and more on the left side, with feeling like *fire*, or as if *burnt;* should compare with *Phytolacca*, which feels as if a red-hot ball was lodged in fauces, is dry, rough, like *Æscul.*, also the congestion is *dark red*, but the soreness is more on the *right* side, and with the dryness and rough feeling, the throat feels large, like a cavern. *Lachesis* should also be compared, as its throat diseases commence on the *left* side and spread to *right*, but remains worse on *left* side, generally it is more from *warm drinks*, and the external throat is excessively sensitive to touch.

In the burning of the throat, which is more from swallowing, *Arnica*, Carbn. s., Kali b., Kali c., and Mag. c. should be studied.

The mucus which is secreted in the throat is of a *ropy* character, and should therefore be compared with Kali bich., Myrica, and Phytol.; this mucus is often of a sweetish taste, and when this symptom is present, care should be taken in studying *Sumbul.*

Often cough is excited from the presence of mucus in the throat, and this condition is peculiar to not only Æsculus, but Atropinum, Gratiola, Kreos., and Senega; if this condition should be present in the morning on rising, study *Amm. brom.*

John V. Allen, M. D.

---

### Ailanthus Glandulosa.

### THROAT—OBJECTIVE.

Inflamed, worse swallowing.
Livid.
Mucus, hawking up of.
" rising of; and yellow matter from.
" great accumulation of; part of which is easily expectorated, while a portion is with much exertion detached in small flakes.
Parotid gland enlarged, tender.
Red (dark), almost purple.
Tonsils, inflamed, with spots of incipient ulceration.
Thyroid gland, enlarged, tender.
Ulcers, tonsils studded with many deep, angry looking; from which fetid discharge exudes.
Ulcers, spreading.

### SUBJECTIVE.

Astringent, sensation as after applying an.
Choking, croupy.
Dry, rough, and scrapy; worse morning.
Ear, pains extend to, when deglutition is painful.
Feeling in, choky, dry, œdematous, thick.
Fullness in the, just above the sternum, and a desire to hawk up something.
Hawking of mucus from.

Hawking constant, with efforts to raise lumps of whitish matter.

Irritability of the.

Neck, swollen feeling of the muscles of the.

Rough, feeling.

Tender, sore, worse swallowing, worse on admission of air.

### AGGRAVATIONS FROM.

Air, on admission of.

Morning, in the.

Swallowing.

### CONCOMITANTS.

Severe headache; dizziness; heat and redness of the face, of mahogany color.

Drowsy, yet very restless and anxious; later insensibility, with muttering delirium; recognizes no one.

Eyes suffused and conjested; startled look when roused; pupils dilated and sluggish; photophobia; vomiting with stupor.

Vomiting violent, sudden, when sitting up.

It is especially indicated or called for in low, adynamic forms of disease; sudden and extreme prostration; torpor; vomiting; pulse small and rapid; purplish skin.

In scarlet fever of a malignant type; the miliary eruption is in patches, with efflorescence between the points of rash of a dark, almost livid color; most on the forehead and face.

Especially suited to women and children more than men; old people least of all; to the asthmatic the odor of flowers is unbearable.

### DIFFERENTIATION.

In this remedy we notice one aggravation in the tender, sore throat, namely, *on admitting air.* Now Cistus can., Merc., and Nux v. have pain in the throat from inhaling cold air, and *Arg. n.* and Hepar when breathing.

Cistus—is better from swallowing, which is the reverse of *Ailanthus.*

*Mercurius.*—Under Merc. we have the tonsils dark-red and studded with ulcers, similar to *Ailanthus*, but there is more salivation even with the painful dryness when Merc. is called for.

*Arg. nit.*—Here again we have the *dark-red* color of the throat, especially of uvula and fauces, but the sensation on breathing is like that of a *splinter* in throat and not the marked soreness of *Ailanthus.*

*Hepar.*—Now we find the *thread* as it were of this remedy, worse from cold air, even in the throat symptoms, but the sensations are as if a fish bone or plug had lodged there.

In the aggravation of throat symptoms in the *morning*—Amm. c., Berberis, Calc-ph., China-sulph., Cistus, Niccolum, may be called, but their respective indications will help to decide.

JOHN V. ALLEN, M. D.

